# Effect of Stent Radial Force on Stress Pattern After Deployment: A Finite Element Study

**DOI:** 10.1007/s11665-014-0913-z

**Published:** 2014-02-26

**Authors:** Alessandro Borghi, Olive Murphy, Reza Bahmanyar, Chris McLeod

**Affiliations:** 1 Institute of Child Health, University College London, London, UK; 2 Institute of Biomedical Engineering, Imperial College London, London, UK

**Keywords:** biomaterials, modeling, superalloys

## Abstract

The present article presents a method for assessing the radial stiffness of nitinol stents. An idealized stent model was created, and its radial stiffness was calculated by means of finite element modeling. The calculations were validated against experimental measurements. The variation of radial stiffness with geometrical dimensions was calculated, and the effect of increasing radial stiffness on endovascular deployment was analyzed. Peak tensile and compressive stresses as well as stent penetration were calculated in the case of an idealized pulmonary artery model having realistic dimensions as well as stiffness. The results of stress calculations were compared with a second set of simulations, where an idealized behavior of the stent (uniform expansion to a theoretical contact diameter) was modeled. The results show how in reality nitinol stents behave in a non-ideal way, having a non-uniform expansion and exerting non-uniform pressure on the contact areas with the artery. Such non-ideality decreases though with the increase in radial stiffness. The radial force alone may be insufficient in describing the stent-artery interaction, and numerical modeling proves to be necessary for capturing such complexity.

## Introduction

The effectiveness of a stent lies in its capability to resist elastic outward recoil of the artery in which it has been implanted: a major indicator of such feature is the stent radial force (RF) (Ref 1), which the manufacturers need to quantify according to international standards (Ref 2, 3) in order to obtain permission to market. Several studies have highlighted a positive relationship between the onset of vascular remodeling and calculated RF (Ref 4, 5); however, such theory is not universally supported (Ref 6). Works in the literature have shown how the mechanical properties of stents highly depend on their geometrical features (Ref 7). On the other hand, in vitro and numerical [finite element modeling (FEM)] methods for stent RF assessment (Ref 8) assume idealized loading conditions (loop strap test and plate crush test) (Ref 9) which are not adequate to analyze the interaction between the stent and the artery. Furthermore, they fail to highlight local stress intensification due to the complex stent geometry. Such complex interaction is well captured in FEM of stent deployment into arterial models, which enables the prediction of the stress pattern on the arterial lumen and provides a tool for comparing the performance of different stent models (Ref 10, 11). This work combines the analysis of RF using conventional methods with FEM analysis of the stress pattern after deployment.

## Methodology

**Fig. 1:**
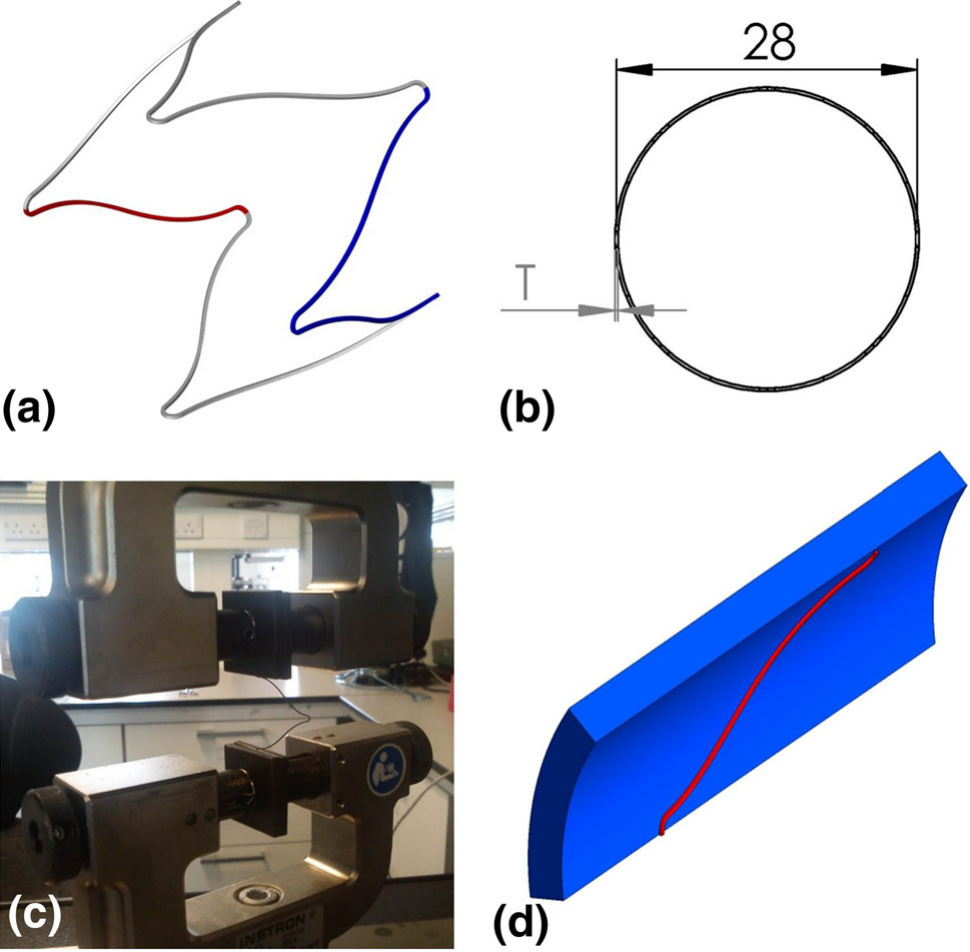
(a) Isometric view of the modified Z stent (in blue, the portion analyzed for mechanical validation; in red, the portion used for FE modeling); (b) dimensions of the stent model, *T* being the thickness (*T* = 0.20 for LOW, *T* = 0.29 for MEDIUM, and *T* = 0.4 for HIGH); (c) nitinol replica of the validation model tested using an Instron® tensile testing machine; (d) 3D FE model of the assembly stent-artery used for FE modeling of stent-artery interaction (in this view, the stent has been deployed) (Color figure online)

To study the effect of varying stent RFs on the arterial stress pattern induced by deployment, an idealized stent CAD model was created and results of RF calculation were analyzed. A modified Z stent was designed using Solidworks®, having a diameter of 28 mm and a length of 21 mm. The stent bars (struts) have a cross section of 0.29 mm × 0.29 mm (this model will be from now on referred as MEDIUM) (Fig. 1).

Finite element modeling, implemented in ANSYS 14.0, was used to analyze the mechanical behavior of the stent. In order to validate the FE results, a planar model of a quarter of the stent was created and its behavior under in-plane axial tension was analyzed using ANSYS. The stent material was modeled as hyperelastic nitinol (*E* = 40 GPa, lower plateau stress = 195 MPa, upper plateau stress = 440 MPa, *ε*_L_ = 0.07, data from Vascotube ®). Both stent and artery models were meshed using 3D hex dominant elements available in ANSYS. Three elements were used across the artery thickness; a finer mesh (0.2 mm maximum dimension for each element) was used for inner and outer surface to improve accuracy of stress calculation. To ensure consistency, the same mesh was used for the artery model in each simulation.

Validation was performed by comparing the FE model with experimental results gained by means of an identically shaped, laser-cut nitinol prototype tested in tension on a tensile testing machine (Instron®) (Fig. 1c). The prototype was tested three times, and results were averaged. Results were compared, and differences were quantified. Two more models of stent were created: both having the same length and diameter but the first of them having strut cross section equal to 0.20 mm × 0.29 mm (LOW) while the second having strut cross section 0.40 mm × 0.29 mm (HIGH). The geometrical dimensions of the models are reported in Table 1.

**Table 1:** Summary of FE models

Model	Stent thickness	Simulation
LOW	*T* = 0.20 mm	Radial crimping for radial force estimation
MEDIUM	*T* = 0.29 mm	Radial crimping for radial force estimation
HIGH	*T* = 0.40 mm	Radial crimping for radial force estimation
*D*_low_	Same as LOW	Free expansion
*D*_medium_	Same as MEDIUM	Free expansion
*D*_high_	Same as HIGH	Free expansion
*I*_low_	Same as LOW	Ideal behavior (displacement control)
*I*_medium_	Same as MEDIUM	Ideal behavior (displacement control)
*I*_high_	Same as HIGH	Ideal behavior (displacement control)

The RF response of the stent model was retrieved numerically by crimping the stent model using a uniform radial displacement on the outer surface. For each stent model, the radial force versus diameter (RF versus D) curve was plotted.

To assess the effect of each stent model deployment into large artery, an idealized artery geometry was created. Due to the symmetry of the structure, only 1/8 of the stent and artery was modeled to simplify the solution and decrease computational time (Fig. 1, in red). The artery was modeled as a hollow cylinder having an inner diameter of 26.6 mm and wall thickness equal to 2.6 mm, which are average dimensions for main pulmonary artery (PA) as reported by Matthews et al. (Ref 12). A section of 42 mm (twice the length of the stent) was analyzed. The artery was modeled as an isotropic material having Young’s modulus *E* = 128 KPa and Poisson ratio *ν* = 0.49 (incompressible). These properties are relative to the pulmonary artery (Ref 12), and the stiffness was calculated by averaging the stiffness values reported for the circumferential and longitudinal direction.

To model stent-artery interaction, the artery was expanded to a diameter greater than the stent by means of external traction and then gradually returned to its resting condition allowing contact with the stent model (in similar way to that reported by Lally et al. (Ref 13)); the contact with the stent was modeled as frictional (*f* = 0.05) (Ref 14). A separate FE model of stent deployment was created for each stent configuration: *D*_low_ having the same stent dimensions as LOW, *D*_medium_ having the same stent dimensions as MEDIUM, and *D*_high_ having the same stent dimensions as HIGH (see Table 1). Wall stress distributions, as well as stent penetration, were analyzed and compared.

**Fig. 2:**
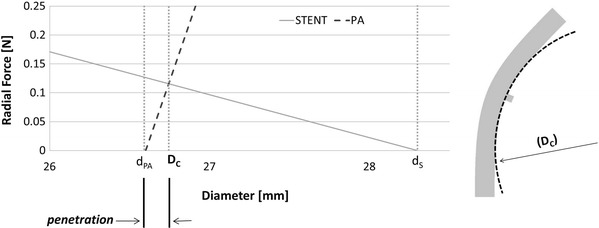
On the left, graphical visualization of interaction between nitinol stent and PA: in its uncrimped configuration, the stent has a diameter equal to *d*_s_; it is then crimped and introduced into the delivery system. Upon release, the stent makes vessel contact once it reaches the diameter of the PA (*d*_PA_). As the RF of the stent exceeds that of the PA, it expands the artery until stress equilibrium is reached (*D*_c_). The penetration is calculated as difference between the artery inner diameter and the contact diameter at equilibrium. On the right, visualization of contact diameter *D*_c_

A simplified method for calculating the final deployment diameter was devised, which relies on the comparison of the stent RF and the elastic response of the artery (Fig. 2). According to Snowhill et al. (Ref 7), the RF of a stent is equivalent to the pressure exerted by the stent on the arterial wall multiplied by the relative cylinder surface area. This assumption is the basis of the main method for RF measurement (acetate/Mylar film method) (Ref 9). As shown by Duerig et al. (Ref 1), the “ideal” operating point of the stent can be visualized by intersecting the radial-force/diameter response of the stent with that of the artery segment. The response of the artery to the internally exerted pressure was calculated from thin-walled cylinder theory (since the ratio between thickness and internal diameter is lower than 10): the hoop force developed in response to internal diameter variation ∆*D*_*i*_ was calculated as (see Appendix)

1 $$F = 2 \uppi \times \frac{{\Delta D_{i} }}{{\left( {D_{i} + t} \right)}} \times E \times t \times L$$

where *t* is the thickness, *E* is the artery’s young’s modulus, and *L* is the stent length. The relationship between diameter and hoop force was plotted in the same graph as the stent RF curves and the working point of each stent configuration were identified (Fig. 4) in terms of contact diameter (*D*_c_) and penetration (Fig. 2). The penetration value for each stent model was used as input parameter for a second model, in which the stent was expanded in displacement control to a diameter equal to its *D*_c_. Three FE models were created, where the “ideal” behavior of each stent model was analyzed (*I*_low_ for LOW stent model, *I*_medium_ for MEDIUM, and *I*_high_ for HIGH). Wall stresses in such case were compared with those which retrieve in the equivalent free expansion (*D*) model.

## Results

**Fig. 3:**
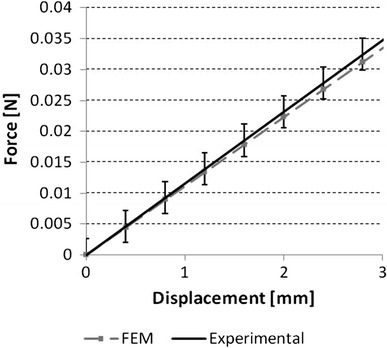
Validation of the finite element model by means of comparison with experimental results

The force-displacement curves were retrieved from the planar FE and compared with experimental data. The force-displacement was analyzed in the range 0-3 mm, equivalent to 21.2% decrease in diameter (28 to 24.4 mm) for the whole stent. A difference of 8.2% in tensile stiffness was found (Fig. 3).

**Fig. 4:**
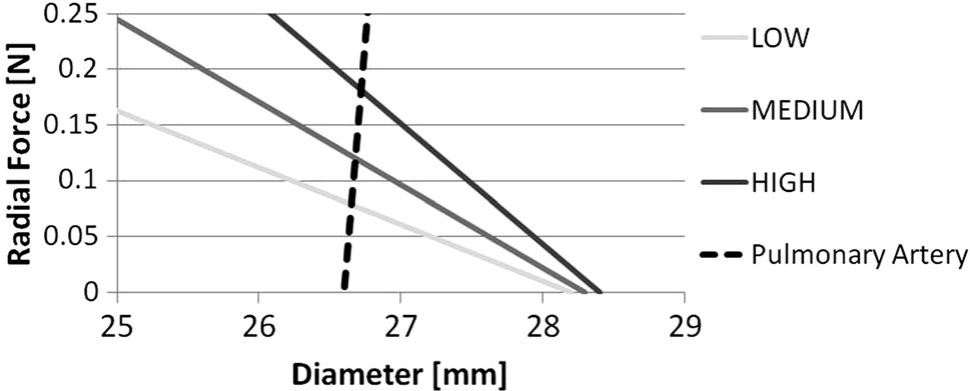
RF vs. D curves for the three stent models as well as PA. The x-axis refers to outer diameter for stent models and inner diameter for PA model

**Fig. 5:**
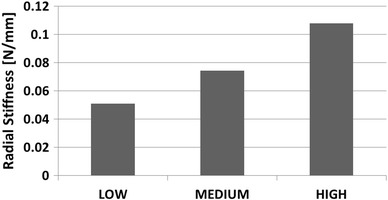
Values of radial stiffness calculated as slope of the RF vs. D curve

Each stent model (LOW, MEDIUM, and HIGH) was then subject to uniform radial displacement, and the reaction force was plotted against external diameter to produce the RF versus *D* response (Fig. 4). The stent radial stiffness was quantified, and the slope of the curves (radial stiffness) was quantified. Figure 5 shows the radial stiffness of the three models.

**Fig. 6:**
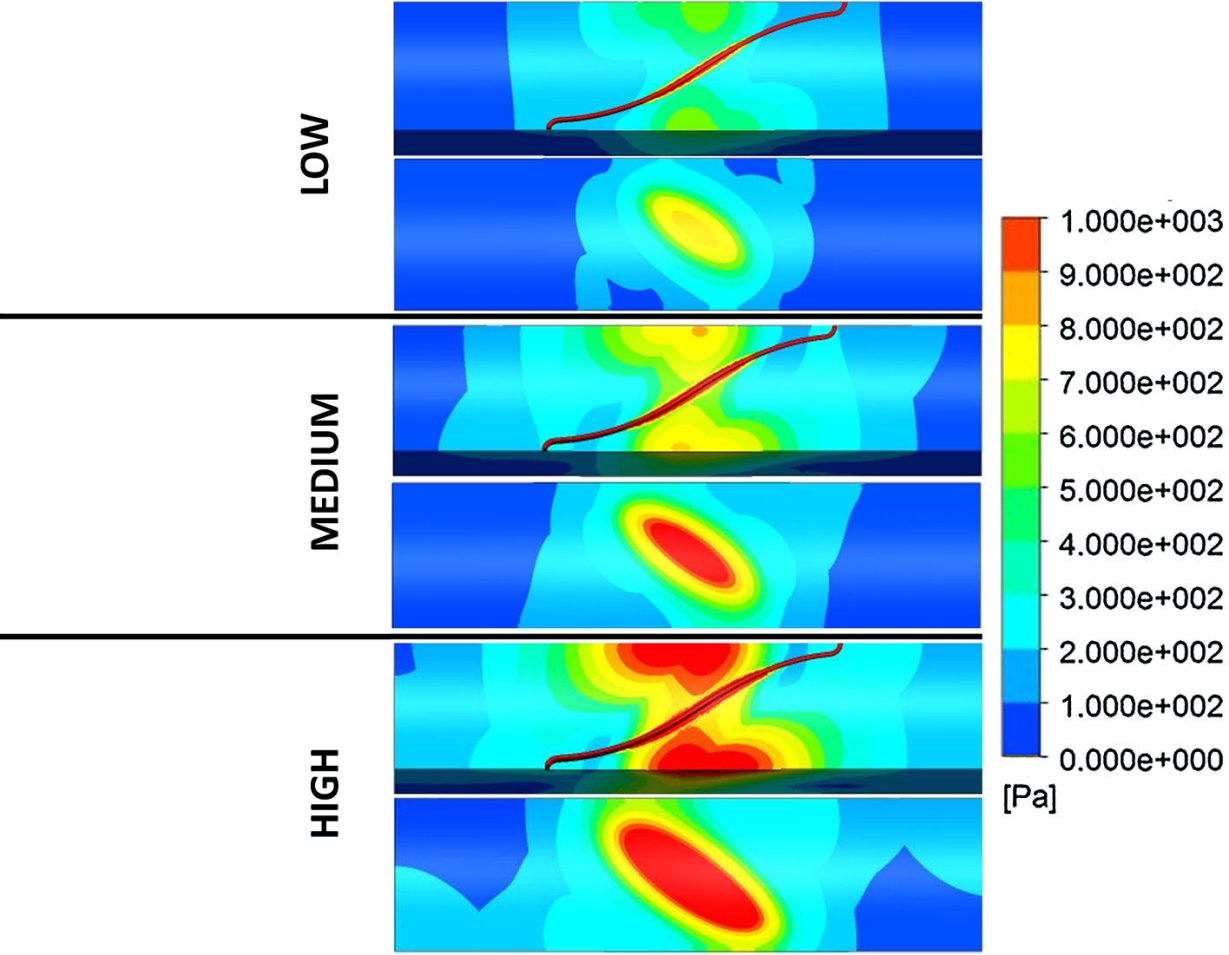
Visualization of stress intensity patterns for the outer (top of each row) and inner (bottom of each row) surface for the three stent models. Units in Pa (Color figure online)

**Fig. 7:**
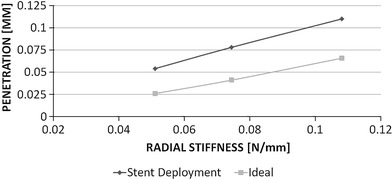
Comparison of stent penetration for the three stent models in the case of stent deployment (*D* model) or theoretical evaluation (*I* model). Units in mm

**Fig. 8:**
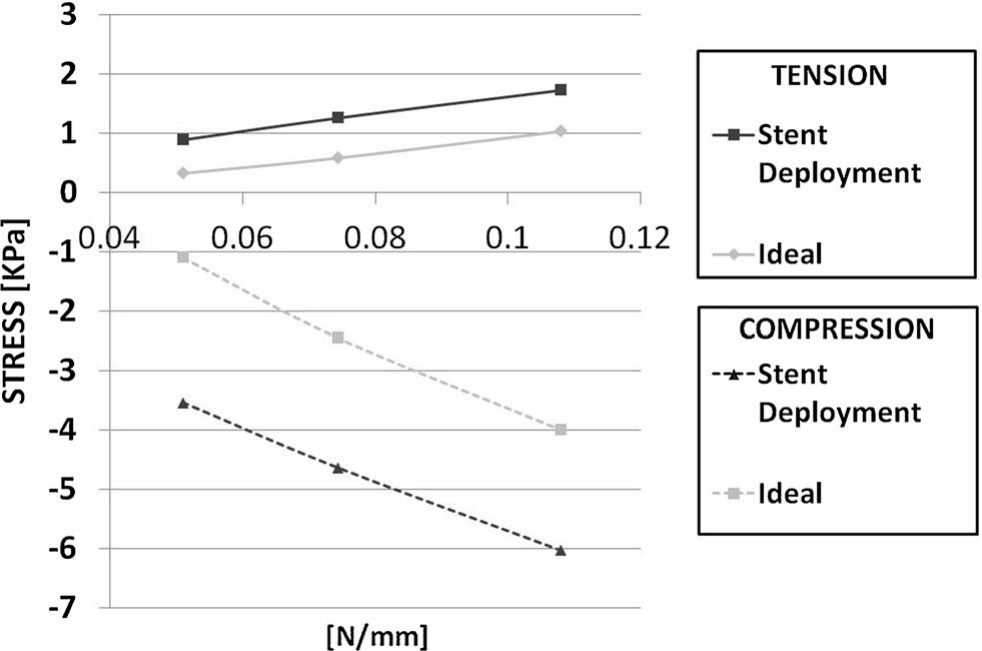
Comparison of peak tensile and compressive stresses for the three stent models in the case of stent deployment (*D* model) or theoretical evaluation (*I* model). Units in KPa

Expansion in an idealized artery was modeled for all three stent models, and the resulting stress patterns were compared. Figure 6 shows a comparison of the stress intensity pattern induced by the different stent models: the penetration depth was calculated as the peak artery radial displacement on the inner surface after the stent deployment (Fig. 7). The values of peak tensile stress (maximum principal stress), occurring on the outside of the artery corresponding to the location of contact with the stent, were quantified and plotted against the values of radial stiffness (Fig. 8). In a similar way, the peak compressive stress (minimum principal stress) occurring on the inner surface of the artery was retrieved and plotted against radial stiffness (Fig. 8).

Both the values of stent penetration and peak stresses increase with the radial stiffness (Fig. 7 and 8). These values were then compared with those calculated theoretically by intersecting the RF curves with the elastic response of a hollow cylinder (*I*_low_, *I*_medium_, and *I*_high_). Figure 7 shows how the theoretical values of the penetration depth of the stent are lower than those calculated from the deployment of the stent. The difference between the models ranges between 51% (*D*_low_ versus *I*_low_) and 40% (*D*_high_ versus *I*_high_).

Peak tensile stresses, calculated as maximum principal stress on the outer surface of the artery, are reported in Figure 8: again, the values found in the stent deployment model (*D*_low_, *D*_medium_, and *D*_high_) are lower than those obtained by calculating the depth of penetration theoretically (*I*_low_, *I*_medium_, and *I*_high_). Similarly, peak negative stresses are larger (in absolute value) for *I* models compared to *D* models.

**Fig. 9:**
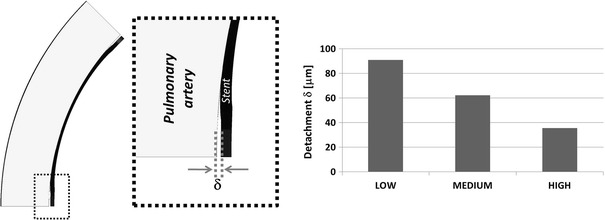
On the left, stent and artery after deployment with zoom on the area where detachment is present; on the right, values of arterial detachment for the three configurations

**Fig. 10:**
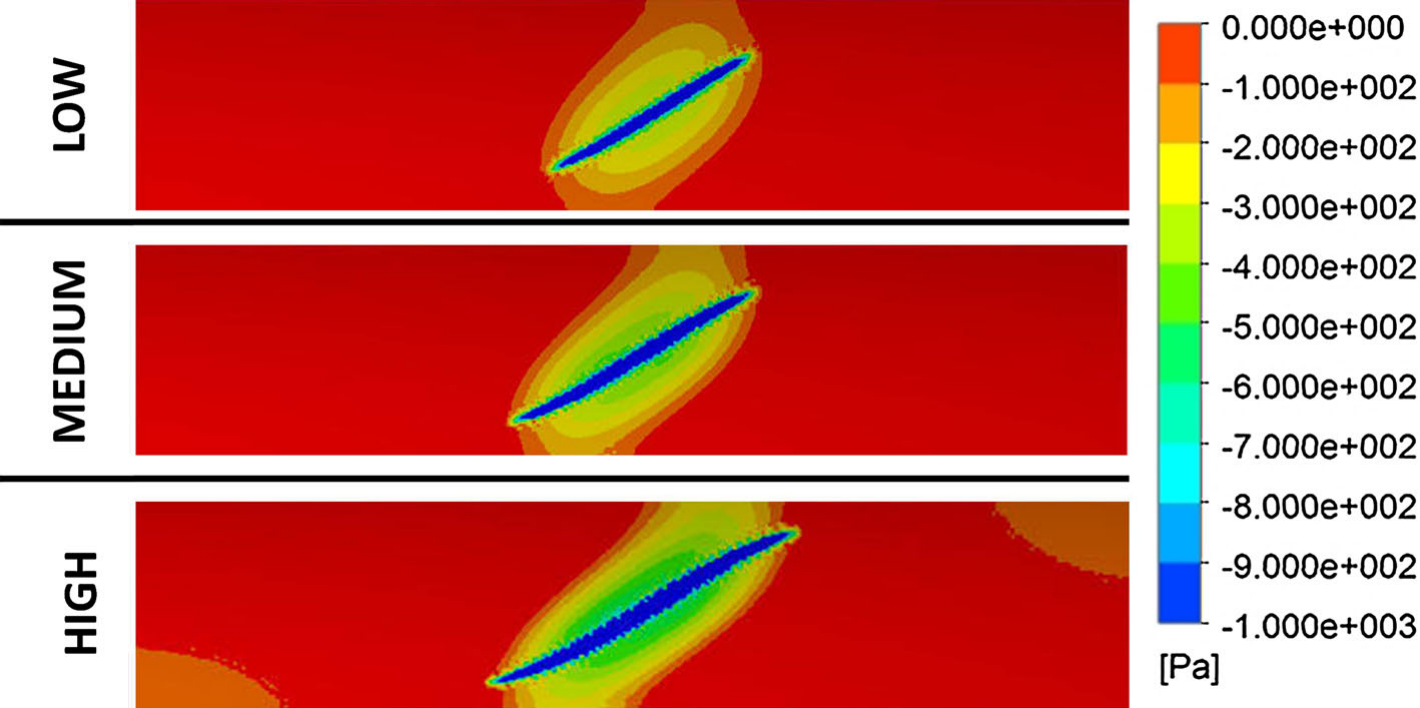
Comparison of stress pattern (minimum principal stress) on the inner wall of the artery deployment in the case of stent deployment (models *D*_low_, *D*_medium_, and *D*_high_). Blue areas refer to high negative stresses where the stent is in contact with the artery (Color figure online)

Since detachment of the stent was observed in all models in correspondence with the stent corners, the amount of detachment (δ) was quantified for each stent (Fig. 9). Figure 10 shows minimum principal stress values on the inner surface of the artery for all the three stent configurations after deployment. The blue region is the area where contact between the stent and the artery occurs. The figure shows how such area increases with stent stiffness and the behavior of the stent becomes more “ideal.”

## Discussion

Variables for comparing commercially available endovascular stents include stent material (stainless steel (Ref 15), cobalt-chromium alloy (Ref 16), biodegradable materials (Ref 17, 18), and shape memory alloys (Ref 19)), and dimensional (percent surface area, foreshortening, and integrity) as well as functional attributes (RF, fatigue performance, and crush resistance) (Ref 3).

The aim of stent characterization is to ensure device safety, correct function after implantation, and prevent adverse vessel remodeling. It has been shown how radial stiffness and restenosis are deeply linked: Cha et al (Ref 4) compared the performance of self-expanding stents having same unconstrained size in a carotid artery canine model. Their results showed that, although larger radial stiffness implied better luminal gain over time, it also caused much larger production of neointima. Their results partially contradicted those of Vorwerk et al. (Ref 6), who using the same commercial stent did not find any difference in neointimal growth between low RF models and high RF models. Freeman et al. (Ref 5) found that by implanting iliac artery stents designed with a predetermined RF (as per Snowhill et al (Ref 7)), they could observe an increase in thickening of the artery with the increase in RF applied and derived the wall stress value at which the arterial wall starts remodeling. The fact that mechanical forces induce proliferation in the pulmonary artery was shown by Kolpakov et al. (Ref 20). By studying excised rabbit PA strips, they showed how increasing stretch, as opposed to increasing hydrostatic pressure, induces media protein synthesis as well as collagen production.

In this paper, a modified z-stent geometry was used for analyzing the effect of varying RF on the wall stress magnitudes and patterns resulting from stent deployment on a model artery. The model was validated by comparing the numerical (FEM) with experimental results from uniaxial tensile testing. Three models of stent having the same diameter but increasing strut thickness were deployed into the model artery, and stress patterns were compared and peak stresses were quantified. The results show how both tensile and compressive peak stresses increase with radial stiffness and how areas of high stress are not necessarily co-located with areas of stent-artery contact. The theoretical working point of each stent, calculated by means of the definition of RF, was graphically calculated for all three stent configurations (with increasing radial stiffness): the deployment diameter was used as input for a model (*I* models) where the stent was simply expanded to that size and the contact with the arterial wall was simulated.

The results show that penetration levels show differences in each pair of models, with percentage difference ranging from 51% in the case of low RF (*D*_low_ versus *I*_low_) to 40% in the case of high RF (*D*_high_ versus *I*_high_). Penetration is an important factor as it provides a measurement of how much the cross section of the artery changes in response to stent deployment. Such parameter has been here quantified thanks to symmetry and was graphically shown in other publications (Ref 10, 21) or quantified in vivo (Ref 22). An ideal stent exerts an uniform pressure on the artery cross section and does not cause localized supra-physiological strains on the vessel; the deformation of the artery also has an effect on the flow pattern within the stented region, which can cause an abnormal biological response (Ref 23).

**Fig. 11:**
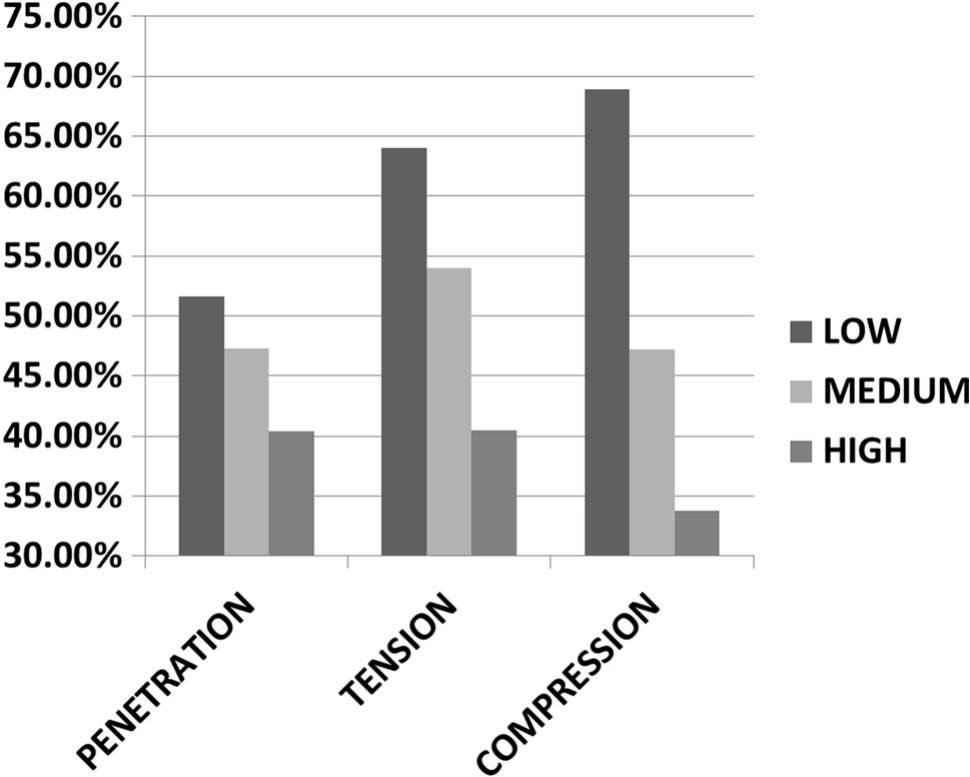
Comparison of penetration and peak tensile and compressive stresses for the three stent models in the case of stent deployment (models *D*_low_, *D*_medium_, and *D*_high_) or idealized behavior (models *I*_low_, *I*_medium_, and *I*_high_)

Differences in peak stress values, both tensile and compressive, show similar trends though percentage differences in stress are higher for the *D*_low_ versus *I*_low_ and lower for *D*_high_ versus *I*_high_ (Fig. 11). At higher RF, the stent has better chances of making the artery conform to its original shape; hence, it retains a section which is closer to a circle: in the *D*_high_ model, the stent after deployment undergoes radial displacement ranging from −0.87 mm to −1 mm, while in the *D*_low_ model the stent undergoes radial displacement from −0.73 mm to −0.88 mm. A larger variation of radial displacement along the stent corresponds to a less circular shape after deployment.

The main limitations of this work lie in the choice of material properties and the modeling of interaction between the stent and the artery. An isotropic material was used to simplify the model and allow the estimation of theoretical values of penetration by means of the thin hollow cylinder theory. The value of stiffness used is taken as the average between longitudinal and circumferential stiffness calculated at the pulmonary artery working point (at strain values of 30%). Although the behavior of the PA is highly non-linear in both human and animal hearts (Ref 12, 24), the properties of the material would vary around this working point.

**Fig. 12:**
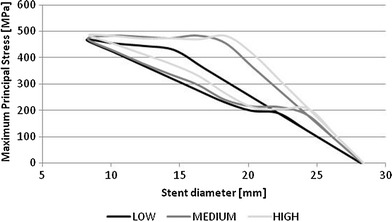
Peak principal stress vs. outer diameter for each stent model. Units in MPa

The interaction between the artery and the stent was modeled by expanding the artery to a diameter larger than the stent by means of an external traction, which was then gradually decreased, allowing contact between artery and stent upon deflation. This method was previously used by Lally et al (Ref 13). It is suitable for elastically behaving stents which respond linearly after balloon expansion but may fail to catch the hysteresis of shape memory alloys, whose behavior depends on the loading history. The stent model used in this work behaves with no hysteresis in the range of diameters analyzed in this work. Figure 12 shows peak principal stress for each stent model when crimped by means of a radial displacement up to 10 mm to show hysteresis due to hyperelasticity. As the graph shows, in the range of diameters involved in this study, all stent models behave in a linear elastic way.

Catheter crimping to a much lower diameter may cause the stresses to exceed the upper plateau stress, causing hysteretic behavior in the response as generally seen in nitinol stents. Results in such case may be quantitatively different, but the comparison would still apply.

In conclusion, the calculation of RF yields information on the interaction between the stent and the artery but it is not sufficient for understanding the localized effects of the deployment in the case of shape memory alloy stents. In the current work, a simplified method was used to quantify radial stiffness, which was previously used in other works (Ref 9, 25). Such method assumes a simplified configuration which does not reflect the in vivo configuration of the stent and fails to catch localized behavior such as stent detachment (Fig. 9, 10). The calculation of radial stiffness with another method (such as rigid body crimp) may yield better results, however, it was shown how—at low RFs—such two methods are equivalent (Ref 25).

The use of FEM is important to understand the correct functioning of the stent and its performance during its lifetime (Ref 26), and RF on its own may be insufficient as in indicator of artery-stent interaction.

## Appendix

From the theory of thin hollow cylinders, the hoop force in the case of open cylinder having thickness, length *L*, and diameter *D* subject to hoop stress *σ*_*t*_ can be calculated as:

$$F = 2\uppi \times \sigma_{t} \times t \times L = 2\uppi \times E \times \upepsilon_{t} \times t \times L = 2\uppi \times \frac{\Delta D}{D} \times E \times t \times L$$

As the mean diameter *D*_*m*_ = *D*_*i*_ + *t* and Δ*D*_*m*_ = ∆*D*_i_ (neglecting the change in thickness):

$$F = 2\uppi \times \frac{{\Delta D_{i} }}{{\left( {D_{i} + t} \right)}} \times E \times t \times L$$
